# Thermal and mechanical high-intensity focused ultrasound: perspectives on tumor ablation, immune effects and combination strategies

**DOI:** 10.1007/s00262-016-1891-9

**Published:** 2016-09-01

**Authors:** Renske J. E. van den Bijgaart, Dylan C. Eikelenboom, Martijn Hoogenboom, Jurgen J. Fütterer, Martijn H. den Brok, Gosse J. Adema

**Affiliations:** 1 Department of Tumor Immunology, Radboud University Medical Center, Geert Grooteplein 28, 6525 GA Nijmegen, The Netherlands; 2 Department of Radiology and Nuclear Medicine, Radboud University Medical Center, Nijmegen, The Netherlands; 3 MIRA Institute for Biomedical Technology and Technical Medicine, University of Twente, Enschede, The Netherlands

**Keywords:** High-intensity focused ultrasound, Tumor ablation, Immunotherapy, Immune adjuvants, PIVAC 15

## Abstract

Tumor ablation technologies, such as radiofrequency-, cryo- or high-intensity focused ultrasound (HIFU) ablation will destroy tumor tissue in a minimally invasive manner. Ablation generates large volumes of tumor debris in situ, releasing multiple bio-molecules like tumor antigens and damage-associated molecular patterns. To initiate an adaptive antitumor immune response, antigen-presenting cells need to take up tumor antigens and, following activation, present them to immune effector cells. The impact of the type of tumor ablation on the precise nature, availability and suitability of the tumor debris for immune response induction, however, is poorly understood. In this review, we focus on immune effects after HIFU-mediated ablation and compare these to findings using other ablation technologies. HIFU can be used both for thermal and mechanical destruction of tissue, inducing coagulative necrosis or subcellular fragmentation, respectively. Preclinical and clinical results of HIFU tumor ablation show increased infiltration and activation of CD4^+^ and CD8^+^ T cells. As previously observed for other types of tumor ablation technologies, however, this ablation-induced enhanced infiltration alone appears insufficient to generate consistent protective antitumor immunity. Therapies combining ablation with immune stimulation are therefore expected to be key to boost HIFU-induced immune effects and to achieve systemic, long-lasting, antitumor immunity.

## Introduction

The immune system is able to detect a wide variety of pathogens and tumor cells, and to distinguish them from healthy host cells. Induction of an adaptive immune response starts with phagocytosis of a pathogen by antigen-presenting cells (APCs), such as dendritic cells (DCs). The phagocytosed antigens are processed into small peptides and presented in major histocompatibility complex (MHC) receptors on their membranes, after which DCs migrate toward lymph nodes (LNs). Further activation signals are required for the establishment of a potent immune response, for instance via recognition of pathogen-associated molecular patterns by pattern recognition receptors (PRR, e.g., Toll-like receptors (TLRs)). DCs subsequently upregulate co-stimulatory molecules, including CD40 and CD80, and present the foreign antigen to T lymphocytes for recognition by their T cell receptors, inducing differentiation of effector and memory CD4^+^ and CD8^+^ T lymphocytes. These cells then perform their effector functions in a concerted manner to eliminate pathogen-infected cells or tumor cells.

In cancer patients, lymphocyte-mediated immunity has failed to prevent primary tumor development. Poor recognition of tumor cells by APCs and the lack of proper activation of these APCs by tumor cells hamper the generation of effective immune effector cells. Also, the presence of immunosuppressive cytokines and that of suppressive tumor-associated cells are common mechanisms by which tumors block the induction and establishment of effective CD8^+^ cytotoxic T lymphocytes (CTLs) or CD4^+^ T helper cells. Over the last few years, however, boosting the immune system through T cell checkpoint blockade, adoptive T cell transfer or vaccination is emerging as an effective treatment modality with clinical benefit for cancer patients [1].

Surgical resection of the primary tumor is still the mainstay of treatment for many cancer patients. However, depending on the tumor type and location of the tumor in the body, this procedure can have severe risks for the patient. During the last few decades, there has been widespread interest in the development and refinement of ablation techniques for local treatment of tumors in a minimally invasive manner. In addition, due to the development of imaging modalities and devices, image-guided tumor ablation is increasingly used for curative treatment, as well as palliative pain treatment. Ablation in its many forms is an attractive alternative treatment option, including for patients otherwise ineligible for surgical resection [2, 3]. Furthermore, tumor debris remaining in situ after ablation may function as an unbiased source of tumor antigens available to the immune system [4]. Possibly, the tumor debris could be used to create an in situ cancer vaccine able to stimulate systemic immune responses toward (micro)metastases already present elsewhere in the body, the so called abscopal effect [5].

The majority of tumor ablation modalities apply energy to cause spatially localized necrosis of tumor cells. Radiofrequency ablation (RFA), microwave ablation (MWA), laser ablation (LA) and high-intensity focused ultrasound (HIFU) employ different sources of energy to rapidly (in seconds or minutes) heat the target region, while cryoablation uses longer (in minutes) cycles of freezing to cause cell death. Complete destruction of the tumor by ablation techniques has several technical difficulties, such as loss of accuracy by respiratory motion or insufficient detection of the tumor borders with different imaging modalities. Incomplete destruction may also occur due to tissue inhomogeneities and asymmetrical heat conduction (heat sink effect). Specifically for HIFU, the presence of gas or bone in the acoustic field results in scattering or absorption of acoustic waves at these interfaces.

**Fig. 1:**
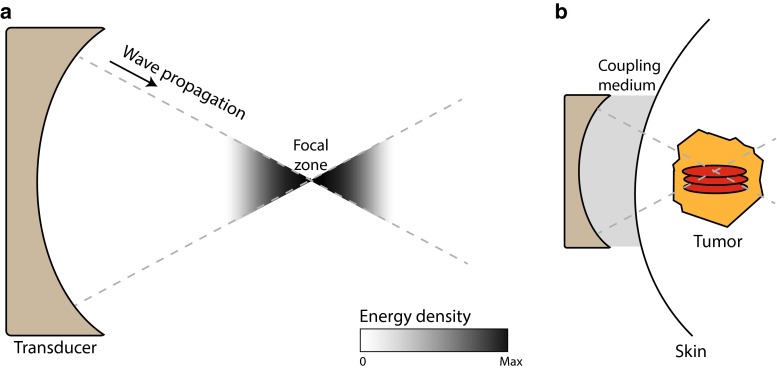
Principles of high-intensity focused ultrasound **a** HIFU ablation employs a transducer, which creates ultrasound beams focused to a single focal zone. The acoustic energy increases near the focal zone. **b** This energy can be used to generate ellipse-shaped thermal or non-thermal lesions in tumors in a noninvasive manner

HIFU is the only completely noninvasive ablation technique available to date and has been applied for the treatment of uterine fibroids and prostate, breast, liver, kidney, bone and brain tumors [6, 7]. HIFU-mediated ablation makes use of a multi-element ultrasound transducer, positioned outside the body or in a cavity, to produce high-intensity ultrasound beams focused to a small region. As the ultrasound beams travel toward the focal zone, the convergence of the acoustic waves leads to an increase in energy density (Fig. 1). In the focal zone, this energy is absorbed by the tissue, elevating temperatures to 60–85 °C in a few seconds. The high temperatures in the focus area lead to coagulation of proteins and fusion of cell membranes, causing necrosis of tumor cells. Heat diffusion leads to a temperature gradient outside the focal zone, where cells do not receive an instantly lethal thermal dose, but are exposed to temperatures over 40 °C. This transition area contains cells suffering from thermal stress. In the days following treatment, the majority of these cells have undergone apoptosis [8]. A similar pattern is observed with RFA, where in the transition zone, defined as the area where cells are exposed to temperatures between 40 and 60 °C, a peak in apoptosis due to hyperthermia-induced mitochondrial damage or impaired membrane function is seen 2 h after treatment [9]. With HIFU, the created lesion is normally ellipse-shaped in the range of a few millimeters. Ablation of larger volumes is therefore achieved by scanning the focal zone through the tumor volume, mechanically or electronically, thereby treating the entire tumor. Real-time visualization of the treatment is performed by either B-mode ultrasound imaging, or magnetic resonance (MR) thermometry [10, 11].

In addition to thermal destruction, HIFU can be used to generate non-thermal effects for disruption of tissue, known as (boiling) histotripsy [12]. Histotripsy and boiling histotripsy are achieved using very short (micro- or millisecond long) acoustic pulses of high intensity (>5 times as high compared to thermal ablation), repeated with a low duty cycle to limit temperature increase. For histotripsy, these high-pressure waves produce changes in the gaseous components in tissues, as bubbles will start to oscillate and burst, causing mechanical damage to tissues at a subcellular level [13]. Boiling histotripsy, which has been applied in mice, uses the formation of a millimeter-sized boiling bubble for mechanical disruption of tissues. The created lesion is very homogeneous, with no visible cellular components, and appears with a sharply demarcated border (<200 µm) between vital and fragmented tissue [8]. For more information on the physical and technical aspects of mechanical HIFU, we refer the reader to [12] and [14].

Poor detection of tumor borders using current imaging techniques and/or outgrowth of micrometastases present prior to ablation elsewhere in the body can lead to incomplete elimination of tumor cells. Local recurrence and distant metastases are currently also major limitations of ablation modalities [10, 15], and these limitations are shared with conventional surgical excision. In this context, it has been proposed by us and others to initiate and/or boost ablation-induced antitumor immune responses by using immunomodulatory agents [16, 17].

Next to lowering the general tumor burden, ablation releases tumor antigens and multiple bioactive molecules such as damage-associated molecular patterns (DAMPs). Combined with general inflammation and immune-regulatory processes of the wound healing response following ablation, this will result in different innate and adaptive immune effects. However, without co-exposure of antigen-loaded APCs to potent stimulation signals, responses generally remain weak. Potent antitumor immunity therefore is rarely generated, as also evidenced by scarce reports of spontaneous regression following ablation [18, 19]. Combining ablation with immunomodulatory adjuvants therefore holds great promise, as providing additional stimuli can overcome immune tolerance and induce DC and T cell activation toward tumor antigen-expressing cells [20]. This concept has been reviewed extensively for RFA, cryoablation and other thermal ablation therapies [21, 22].

HIFU is a relatively new treatment modality with high potential. This review aims to summarize the immune effects after thermal and mechanical HIFU tumor ablation. Furthermore, we will describe parallels with other ablation methods and discuss the future perspectives of combination treatments with ablation to generate effective antitumor immunity.

## HIFU-generated tumor debris

### Tumor antigens

HIFU ablation of tumor cells will lead to either coagulative necrosis (i.e., thermal HIFU) or subcellular fragmentation (i.e., mechanical HIFU). The resulting depot of damaged tumor cells will remain in situ, and tumor antigens present in this depot can be captured by tissue-resident phagocytic cells, such as DCs, that subsequently migrate toward tumor-draining lymph nodes (TDLNs). Alternatively, tumor antigens may passively enter the circulation or lymphatics and be transported to LNs where they can be taken up by LN-resident DCs. The in situ tumor debris will contain all tumor antigens in a (partially) denatured or non-denatured state, depending on the temperatures reached in the focal zone. It has been shown in a cryoablation model that the presence of the depot is essential for the creation of tumor-specific immune responses. There, protection against a rechallenge with the same tumor was attenuated when the tumor debris was excised shortly after ablation, indicating the importance of release of antigens from the depot [23]. Additionally, CT26 colon adenocarcinoma-bearing mice treated with RFA followed by surgical excision (1 week apart) showed a significantly delayed tumor outgrowth after rechallenge 150 days later, compared to non-tumor-experienced naïve mice inoculated with the same tumor cell dose [24]. Also after RFA and cryoablation of B16 melanomas in mice, it has been observed that DCs are able to readily internalize tumor antigens from the tumor depot during the first 2 days, with around 10 % of DCs present in LNs becoming positive for the tumor-derived antigen [25]. These data indicate that the presence of tumor debris is necessary to evoke an antitumor immune response and that ablation is effective in obtaining antigen-loaded DCs in TDLNs.

The goal of personalized cancer therapy hinges on the discovery of suitable antigens giving rise to epitopes present in an individual tumor for the creation of tumor vaccines, recently coined as the HLA ligandome [26]. These vaccines can contain mutated neoantigens, overexpressed self-antigens or tissue-specific proteins for cell types not essential for survival of the patient. In the last few years, several studies have emphasized the importance of recognition of tumor-mutated neoantigens by immune cells [27, 28]. Tumors that are considered as highly immunogenic (i.e., melanoma and lung cancer) have higher rates of somatic mutations, which lead to additional recruitment of neoantigen-specific immune cells [29]. Furthermore, CD8^+^ tumor-infiltrating lymphocytes reactive to clonal tumor neoantigens were identified in early stage non-small cell lung cancers. More strikingly, T cells recognizing clonal neoantigens were detected in patients with good clinical outcomes, while poor responders showed enrichment of T cells recognizing only subclonal neoantigens. These data suggest that immune cells targeting clonal neoantigens play a key role in antitumor protection [30]. In principle, in situ tumor destruction techniques will create an unbiased tumor antigen source in which all types of antigens are present, including mutated neoantigens.

Few studies have investigated the presence of tumor antigens in HIFU-generated tumor debris by making use of mAbs recognizing tumor antigens. One such study demonstrated that in several breast cancer patients, some tumor markers, such as CD44v6 and matrix metalloproteinase-9, were completely absent in immunohistochemically stained tumor biopsies after thermal HIFU ablation, while other tumor antigens could be detected to varying degrees in the HIFU-induced lesion [31]. A common difficulty of antibodies detecting these antigens is the variation in their specificity, and the degree in which they recognize (partially) denatured tumor antigens. Following RFA of colorectal liver metastases, patients show an initial increase of carcinoembryonic antigen (CEA, a tumor antigen), while after surgical resection, the levels of CEA drop rapidly due to elimination of the tumor load. After the initial increase following RFA, levels of CEA slowly drop to background levels over time. This suggests a gradual release of tumor antigens from the in situ depot into the circulation, which can subsequently be taken up by immune cells [32]. Another issue in comparing data from the different HIFU studies is the lack of detail in treatment description (see also Table 1). More research is needed using detailed HIFU protocols to elucidate the nature of tumor antigens present in the debris after thermal and mechanical HIFU ablation and the kinetics of their release into the circulation.

**Table 1:** Overview of described immune effects after high-intensity focused ultrasound tumor ablation in animal studies

Authors	Year	Tumor and animal models	Treatment parameters	Main findings	Additional observations
Chapelon et al. [69]	1992	Dunning R3327 adenocarcinoma in Fischer Copenhagen rats	Frequency: 1 MHz Acoustic intensity: 300–2750 W/cm^2^ Exposure: 3–10 s	No recurrence of primary tumor or appearance of metastases in 14 % (Study 1) and 64 % (Study 2) of HIFU-treated animals	Lower metastatic rate in treated animals (16 vs. 28%)
Yang et al. [50]	1992	C1300 neuroblastoma in male Ajax mice	Frequency: 4 MHz Acoustic intensity: 550 W/cm^2^ Exposure: 5 s per location	Significant inhibition of tumor growth in mice treated with a thermal HIFU, compared to untreated mice	Reduced tumor growth of a secondary tumor on the contralateral side after thermal HIFU of the primary tumor
Hu et al. [52]	2007	MC-38 adenocarcinoma in C57BL/6 mice	Frequency: 3.3 MHz Mechanical: Acoustic intensity: P+ 31.7/P− 12.5 MPa, Exposure: 30 s at 2 % duty cycle Thermal: Acoustic intensity: P+ 12/P− 6.7 MPa, Exposure: 3 s	Thermal and mechanical HIFU increased CD11c-positive cell infiltration in tumors and accumulation of DCs in TDLNs. The antitumor effects were stronger with mechanical HIFU compared to thermal HIFU	Thermal and mechanical HIFU provide protection against subcutaneous tumor rechallenge
Xing et al. [53]	2008	B16F10 melanoma in female C57BL/6 mice	Frequency: 3.3 MHz Mechanical: Acoustic intensity: P+ 31.7/P− 12.5 MPa, Exposure: 30 s at 2 % duty cycle Thermal: Acoustic intensity: P+ 12/P− 6.7 MPa, Exposure: 3 s	Increased cytotoxicity of CTLs when thermal or mechanical HIFU treatment was performed 2 days before amputation of the tumor-bearing leg	Amputation of the tumor-bearing leg 2 days after thermal or mechanical HIFU resulted in a decreased metastasis incidence rate
Chida et al. [70]	2009	Sarcoma-180 in male ICR mice	Frequency: 3 MHz Acoustic intensity: 10 W/cm^2^ Exposure: 10 s	A significant reduction in tumor growth and increased survival of animals were observed after a single shot with HIFU, compared to untreated animals	Significantly higher numbers of TRAP-, CD4- and CD8-positive cells were present in tumors after HIFU treatment
Deng et al. [49]	2010	H22 hepatocellular carcinoma in C57BL/6 J mice	Frequency: 9.5 MHz Acoustic power: 5 W Exposure: 180–240 s	DCs loaded with HIFU-ablated tumor lysate induced significantly higher cytotoxicity and IFN-γ and TNF-α secretion by CTLs against H22 cells, than DCs loaded with untreated tumor lysate	
Zhang et al. [48]	2010	H22 hepatocellular carcinoma in C57BL/6 J mice	Frequency: 9.5 MHz Acoustic power: 5 W Exposure: 180–240 s	Injection of HIFU-treated tumor lysate results in an increase in tumor-specific cytotoxicity of CTLs and a significant decrease in tumor growth, compared to an injection of untreated tumor lysate	Culturing bmDCs in the presence of HIFU-treated tumor lysate slightly increased CD86, CD80 and MHCII expression and IL-12 and IFN-γ secretion, compared to untreated tumor lysate
Huang et al. [54]	2012	RM-9 prostate cancer in C57BL/6 mice	Frequency: 3.3 MHz Acoustic intensity: P+ 32/P− 10 MPa Exposure: 20 s at 2 % duty cycle	Mechanical HIFU, followed by resection of the tumor, inhibits growth of rechallenged tumors, increases CTL numbers in spleen and TDLN and down-regulates STAT3 levels in the tumor	
Liu et al. [55]	2010	MC-38 adenocarcinoma and B16 melanoma tumors in C57BL/6 mice	Frequency: 3.3 MHz Acoustic intensity: P+ 19.5/P− 7.2 MPa Exposure: 4 s	Enhanced infiltration of DCs into tumor tissue in a sparse-scan HIFU treatment regime compared to a dense-scan regime	Tumor cells heated to <55 °C in the periphery of a lesion induce more maturation of DCs than tumor cells heated to >80 °C
Xia et al. [51]	2012	H22 hepatocellular carcinoma in female C57BL/6 J mice	Frequency: 9.5 MHz Acoustic intensity: 5 W Exposure: 220 s total time	A significant increased cytotoxicity of CTLs and a significant increase in IFN-γ and TNF-α secretion by CTLs was observed after thermal HIFU ablation, compared to untreated controls	A significant increased number of activated tumor-specific CTLs after HIFU treatment, compared to untreated controls

*HIFU* High-intensity focused ultrasound, *CTLs* cytotoxic T lymphocytes, *TDLN* tumor-draining lymph node, *P*+ peak-positive pressure, *P*− peak-negative pressure, *DCs* dendritic cells

### Danger signals

After thermal ablation, a lesion of coagulative necrosis is formed, as well as a transition zone of cells undergoing apoptosis at a slower rate due to heat stress [33, 34]. On the other hand, mechanical HIFU ablation leads to cellular fragmentation with only a minimal temperature increase [35, 36]. There have been many studies trying to correlate the type of in vivo cell death to immunogenicity, and the current consensus is that both apoptosis and necrosis can be immunogenic, depending on the release of factors such as calreticulin or heat-shock proteins (HSPs) [37, 38]. At present, the occurrence of such factors and the resulting immunogenicity are still poorly defined for the various HIFU treatments. PRRs on the cell surface of innate immune cells, such as the highly conserved TLRs, are able to discern microbial molecular patterns. However, TLRs are also able to bind a range of endogenously derived self-molecules released in response to cellular damage, known as DAMPs. The binding of DAMPs to PRRs on innate immune cells promotes intracellular signaling cascades, leading to the production of inflammatory cytokines, chemokines and type 1 IFNs. These factors regulate inflammatory responses and coordinate the development of immunity or tolerance to the antigens present [39]. Ablation itself will lead to a physiological wound healing response as a consequence of internal injury. Wound healing is a complex phenomenon comprised of different discrete stages, each predominated by different cytokines and cell types. Some of the initial stages appear more inflammatory, while the tissue regeneration stage involves immune-regulatory cytokines, like TGF-β, that may be more anti-inflammatory or immune suppressive. In conclusion, ablation results in the release of a pleiotropic mixture of signals, including immune stimulatory and immune inhibitory signals.

So far only a limited number of studies have investigated the release of immune stimulatory signals following HIFU ablation. Thermal or mechanical HIFU treatment of MC-38 colon adenocarcinoma cells in vitro resulted in a rapid release of endogenous DAMPs, such as HSP-60 and ATP, from the damaged tumor cells [40], from which the latter can act as chemoattractant for DCs [41]. Subsequently, in vitro incubation of DCs or macrophages with this supernatant resulted in an upregulation of co-stimulatory molecules on their surface (CD80 and CD86), as well as an increased secretion of IL-12 by DCs and an elevated secretion of TNF-α by macrophages. The stimulatory effect was more pronounced by mechanical HIFU treatment compared to thermal HIFU treatment [40]. In addition, it was shown that HIFU could induce HSP-70 and HSP-27 expression in vitro [42, 43]. These results are extrapolated to the in vivo situation, where the ongoing wound healing response is present, confirming the upregulation of HSP-70 in the skin of mice after thermal HIFU treatment [44]. Clinical evidence demonstrates upregulation of HSP-27, HSP-72 and HSP-73 after HIFU treatment in prostate cancer [43, 45]. This upregulation was specifically seen at the border zone of the HIFU-induced lesion [45]. In breast cancer patients treated with thermal HIFU, HSP-70 was found to be upregulated in the tumor debris [31]. These results are similar to results obtained from other ablation methods, where RFA-treated B16 melanomas became highly positive for HSP-70 and glycoprotein 96 (gp96) [46]. Similarly, Haen et al. also demonstrated a significant systemic release of HSP-70 into the serum one day after RFA treatment of lung, liver and kidney malignancies [47]. Furthermore, they observed a better clinical outcome in the group with significant HSP-70 release compared to the group without increase in HSP-70 serum levels. However, this was investigated in a small cohort with large variation, so these findings need to be confirmed in independent studies [47]. Most studies looking for HIFU-induced DAMP release have focused on HSPs, future investigations should include a broader range of DAMPs, and need to define the impact of these individual factors on the immunological outcome. Furthermore, the release and the effect of immune inhibitory signals following ablation should also be examined.

In conclusion, tumor ablation will lead to formation of an in situ antigen depot containing all tumor antigens, including mutated neoantigens, which can be processed and presented by APCs. Furthermore, ablation will lead to the release of DAMPs that potentially could activate cells from the innate immune system, such as DCs. However, the kinetics of release of tumor antigens and DAMPs from the depot after thermal or mechanical HIFU require further investigation. In current HIFU literature, however, comparisons between different HIFU treatments are complicated by lack of details in the treatment description, as well as classification of the acoustic field generated and the temperature in the lesion (see also Table 1). Definition of the molecular fingerprint of different ablation approaches may help to predict whether the ablation-induced inflammation will lead to tolerance or a productive antitumor immune response.

## HIFU-induced immune effects

### Experimental evidence

Despite the reported enhanced presence of key immunological correlates following ablation, strong immune responses have not been observed after tumor ablation as monotherapy. Possibly, ablation-induced immunological activation and wound healing responses, triggered within the same time frame, serve more to regulate and maintain immunological tolerance toward the damaged tissue. Increasing evidence indicates that HIFU-induced tumor ablation can modulate antitumor immunity (summarized in Table 1). Zhang and colleagues investigated, using H22 hepatocellular carcinoma (HCC) bearing mice, whether tumor debris could be an effective vaccine to elicit tumor-specific immune responses [48]. The HIFU-generated tumor vaccine significantly increased CTL cytotoxicity and induced enhanced activation of immature DCs. Mice immunized with the HIFU-generated tumor vaccine showed inhibited tumor growth after a subsequent H22 tumor challenge compared with control mice. Similarly, lysate from thermal HIFU-treated H22 tumors was shown to induce maturation of DCs [49]. Injection of DCs loaded with HIFU-ablated tumor into naïve mice resulted in increased CTL cytotoxicity and inhibited tumor growth of a H22 tumor challenge compared to controls [49]. Alternatively, tumor eradication by thermal HIFU treatment was shown to significantly reduce tumor growth of rechallenged tumors in a neuroblastoma model [50]. Furthermore, thermal HIFU ablation of H22 HCC tumors resulted in increased cytotoxicity of CTLs, along with a significant increase in IFN-γ and TNF-α secretion, compared to untreated controls [51]. Additionally, a significant increase in the number of tumor-specific CTLs in the HIFU-treated cohort was seen. Adoptive transfer of these HIFU-activated CTLs was shown to evoke potent antitumor immune responses in tumor-bearing mice in terms of survival benefit and tumor regression [51]. Similar results regarding CTL activation were obtained from mice bearing B16F10 melanomas and MC-38 colon adenocarcinomas [52, 53]. Interestingly, mechanical HIFU ablation of B16F10 tumors was slightly more potent in activating CTLs compared to thermal HIFU ablation [53]. Furthermore, HIFU treatment, thermal and mechanical, of MC-38 adenocarcinomas resulted in enhanced infiltration of CD11c^+^ DCs into tumors and subsequent migration to TDLNs [52]. Again, these effects were more pronounced in mechanical HIFU-treated mice compared to thermal HIFU-treated mice [52]. Mechanical HIFU ablation of RM-9 prostate tumors followed by resection 2 days later resulted in increased numbers of CD8^+^ cells in spleens and TDLNs, and these cells exhibited higher tumor-specific cytotoxicity. The cumulative survival of this dual treatment cohort was found to be statistically higher than that in the surgery group [54]. These effects are similar to several studies using RFA treatment, where increased numbers of tumor-reactive CTLs were observed 24 h after RFA treatment, with increased CD3^+^ cells infiltration in the transition zone (reviewed in [22]). For thermal HIFU, a treatment regime where each thermal lesion does not overlap with its neighbors has been recommended, as it has been shown that DCs accumulate mostly in the periphery of a lesion, where tumor cells are exposed to temperatures of <55 °C [55]. This observation implies that sparse-scan thermal treatment regime could be more potent in stimulating immune effects, indicating the importance of optimization of the HIFU scan strategy for optimal tumor ablation and stronger immune responses.

Taken together, these data suggest that HIFU ablation alone does significantly influence the immune system, but that the overall antitumor immune response is insufficient. Therefore, it has been suggested that different immune stimuli can help boost ablation-induced immune effects. For cryoablation, it is known that in vivo proximity of tumor antigen and immune stimulus (in these studies; TLR-9 agonist CpG-ODN) in place and time is essential for optimal immune activation. Efficacy of cancer immunotherapy was strongly increased only when CpG-ODN was administered peritumorally immediately after cryoablation [56, 57]. Furthermore, a combination strategy of cryoablation, adoptive transfer of DCs and CpG-ODN resulted in reduced tumor growth, metastasis formation and protection against recurrence of Lewis lung carcinoma [16].

In the B16 model, RFA and cryoablation have also been combined with a checkpoint blockade antibody directed against CTLA-4, resulting in increased numbers of tumor-specific T cells with increased IFN-γ secretion potential, and protection against outgrowth of tumor rechallenges [25]. Later, these results were confirmed in a mouse model of prostate cancer [58]. In this latter study, cryoablation of primary tumors alone also did not affect growth of secondary tumors. Systemic effects were only achieved by combining cryoablation with CTLA-4 blockade, which led to high infiltration of CD4^+^ and CD8^+^ T cells, as well as an increase in effector T cell/regulatory T cell ratio in secondary tumors [58]. Analyses of the effect of multiple other adjuvants in combination with ablation have implicated saponins, a new class of non-microbial adjuvants, as being particularly potent [17]. The data have shown that saponins combine effectively with cryoablation, leading to more efficient uptake of tumor antigens by CD11c^+^ DCs in TDLNs, enhanced cross-presentation and activation, compared to cryoablation alone [17].

### Clinical evidence

As shown in Table 2, clinical results also reveal modulation of the immune system after thermal HIFU ablation. Mechanical HIFU tumor ablation has not yet been performed in a clinical setting. So far, several patients with various solid malignancies, who had an abnormal CD4^+^/CD8^+^ T cell ratio prior to HIFU treatment, had their CD4^+^/CD8^+^ ratio revert to normal 1 week after ablation [59, 60]. In a different study, ten out of 15 patients with late-stage pancreatic carcinomas showed significantly increased NK cell activity after HIFU treatment, as well as a trend toward more CD3^+^ and CD4^+^ cells in peripheral blood was observed [61]. Thermal HIFU ablation has also been shown to increase the infiltration of DCs, macrophages and CD3^+^, CD4^+^ and CD8^+^ lymphocytes in the margins of induced lesions in breast tumors, compared with untreated tumors [62, 63]. These systemic cellular effects are only present in a subset of patients, and effective tumor-specific immune responses are not observed. These results are comparable to preclinical data, where increases in immune cell infiltration and activation can be seen after HIFU treatment.

**Table 2:** Overview of described immune effects after high-intensity focused ultrasound tumor ablation in clinical studies

Authors	Year	Patient information	HIFU parameters	Main findings	Additional observations
Rosberger et al. [59]	1994	5 patients with choroidal melanoma	Exposure: >50 °C for 5 min	CD4^+^/CD8^+^ ratio reverted to normal after HIFU in 2 of 3 patients with an abnormal CD4^+^/CD8^+^ ratio	
Wang et al. [61]	2002	15 patients with late-stage pancreatic carcinoma	Frequency: 1.1 MHz Acoustic power: 500–1600 W Exposure: 30–80 s per location	A significant increase in the activity of NK cells after HIFU treatment	Nonsignificant increase in CD3^+^ and CD4^+^ T cells in 66 % of patients (10/15)
Wu et al. [71]	2003	23 female patients with biopsy-proven breast cancer	Frequency: 1.6 MHz Acoustic intensity: 5000–15,000 W/cm^2^ Exposure: 30–180 min total time	HIFU-treated tumors showed significant decrease in PCNA, CD44v6, MMP-9 and erbB2 mRNA levels	
Kramer et al. [45]	2004	6 patients with prostate cancer	Frequency: 4 MHz Acoustic intensity: 1260–2000 W/cm^2^ Exposure: 4 s per location	A significant upregulation of HSP-72 and -73 at the border zone of HIFU-induced thermal lesion in prostate cancer patients	
Wu et al. [60]	2004	16 patients with solid malignancies	Frequency: 0.8 MHz Acoustic intensity: 5000–20,000 W/cm^2^ Exposure: 2.5–8 h total time	A significant increase in CD4^+^ T cells after HIFU treatment	CD4^+^/CD8^+^ ratio reverted to normal after HIFU in 3 patients with an abnormal CD4^+^/CD8^+^ ratio
Zhou et al. [66]	2008	15 patients with various solid malignancies	Frequency: 0.8–1.2 MHz Acoustic intensity: 140–260 W Exposure: 4–39 min total time	A significant decrease in serum VEGF, TGF-β1 and -β2 cytokine levels after HIFU treatment	
Wu et al. [31]	2007	23 female patients with biopsy-proven breast cancer	Frequency: 1.6 MHz Acoustic intensity: 5000–15,000 W/cm^2^ Exposure: 45–150 min total time	HSP-70 expression was detected on the ablated cancer cells in all patients treated with HIFU	No expression of CD44v6, MMP-9 and PCNA in HIFU-treated tumors
Lu et al. [62]	2009	23 female patients with biopsy-proven breast cancer	Frequency: 1.6 MHz Acoustic intensity: 5000–15,000 W/cm^2^ Exposure: 45–150 min total time	A significant increase in CD3^+^, CD4^+^ and CD8^+^ T lymphocyte infiltration in the tumor, compared to controls	Increased numbers of NK cells and FasL+, granzyme+, perforin+ TILs found in HIFU-treated tumors
Xu et al. [63]	2009	23 female patients with biopsy-proven breast cancer	Frequency: 1.6 MHz Acoustic intensity: 5000–15,000 W/cm^2^ Exposure: 45–150 min total time	A significant increase in infiltration and activation of macrophages and DCs in HIFU-treated tumors, compared to controls	
Wang et al. [64]	2013	120 patients with uterine fibroids	Frequency: 0.8 MHz Maximum acoustic power: 400 W Exposure: not stated	Serum levels of IL-6 and -10 increased after HIFU treatment	IL-2 serum levels remained stable in HIFU-treated patients, compared to the patients receiving surgical resection where the IL-2 levels decreased

*HIFU* High-intensity focused ultrasound, *DCs* dendritic cells, *TILs* tumor-infiltrating lymphocytes, *NK cells* natural killer cells

Evaluation of immune-related cytokines showed increases in the Th2 cytokines, IL-6 and IL-10, in serum after ablation, although it was not determined what cell type secretes these cytokines [64, 65]. The increase in IL-6 and IL-10 in plasma levels was observed within 48 h using different ablation techniques, where cryoablation induced greater changes than heat-based ablation [65]. On a serum level, a significant decrease in the immunosuppressive cytokines, including vascular endothelial growth factor (VEGF), TGF-β1 and -β2, was measured after HIFU treatment in patients with various solid malignancies, suggesting that HIFU may reduce immunosuppression [66]. In a retrospective study, RFA treatment of colorectal cancer liver metastases increased T cell infiltration, as well as PD-L1 expression in primary colon tumors [67]. The authors confirmed these findings in a CT26 tumor-bearing mouse model. Furthermore, they observed that while RFA of a tumor can induce strong T cell responses in the distant tumors, these tumors quickly overcame this by inhibiting T cells via upregulation of PD-L1/PD-1 expression. In this setting, combining RFA with anti-PD-1 antibodies showed stronger T cell responses and resulted in significantly prolonged survival of the tumor-bearing mice [67].

In conclusion, immune effects after tumor ablation alone consist mostly of increased infiltration of immune cells, including innate and adaptive immune cells, in the destroyed tumor tissue, which is observed in experimental and clinical setup. In several murine tumor models, enhanced DC and CTLs activities are observed. In man, the results revealed mainly changes in the secretion of inflammatory, as well as immunosuppressive cytokines. Systemic protection after HIFU has not been observed frequently, which is in line with studies using cryoablation or RFA. There, systemic effects were only achieved when ablation was combined with immune adjuvants, including checkpoint blockade antibodies.

## Conclusion and perspectives

HIFU is an important development toward a completely noninvasive ablation treatment. Thermal and mechanical HIFU ablation is being used in various pre-clinical settings for different solid malignancies. Thermal HIFU ablation is applied in various clinical settings, while for mechanical ablation the first clinical trial is being performed. Although preliminary data do suggest that immune effects occur after HIFU ablation, such as increased infiltration and cytotoxicity of CTLs, no potent tumor-specific immunity has yet been convincingly demonstrated. Despite the obviously changing immunological parameters, the minor decreases in tumor growth after rechallenge, and inconsistent decrease in metastasis formation after HIFU alone, do not support the induction of strong antitumor immune responses. Data retrieved from HIFU studies so far are in line with other ablation technologies and strengthen the notion that ablation should be combined with immunomodulatory adjuvants to boost antitumor immune responses. Combination strategies could lead to an in situ tumor vaccine, where tumor antigens are released from the tumor debris and taken up by APCs, while the immunotherapeutic compound helps activate immune cells and overcome immunosuppression. Only in such a scenario, long-lasting systemic immunity against the tumor can be expected. Further studies will elucidate by what mechanism HIFU induces or enhances immune responses and what immunomodulatory adjuvants synergizes best with each type of ablation in different cancer types. Selecting the best ablation-immune stimulation combination will be key to boost HIFU-induced immune effects and to achieve consistent protective antitumor immunity.

The effects of tumor ablation are multifold: (1) the destruction of tumor mass, lowering tumor burden and (2) the release of tumor antigens, making them available for uptake by APCs. The treatment itself will lead to (3) the release of DAMPs and (4) the induction of a physiological wound healing response. Ablation will lead to creation of an in situ antigen depot containing all types of tumor proteins. Ablation of tumors at temperatures above 65 °C leads to denaturation of proteins. This can affect immune responses in opposing ways as high temperatures denature immune activating signals, such as danger signals like HSPs, as well as immune suppressive signals such as TGF-β or IL-10. Depending on the tumor microenvironment, it may be more important to remove immune suppressive signals or maintain danger signals using, respectively, thermal ablation or mechanical ablation. Furthermore, availability of tumor antigens from the tumor debris may be different between thermal and mechanical HIFU. The state of blood vessels in/near the treated area should be considered as well, since the majority of immune cells will reach the induced lesion via the circulation. More experiments looking closely at the optimal treatment regime for a given cancer patient are needed to achieve this. In current literature, however, the treatment description, as well as classification of the acoustic field generated, is often lacking details (see also Table 1). Furthermore, it is important to know the temperatures reached and whether a more sparse- or dense-scan treatment is used. A standardized framework of treatment description, such as proposed previously, could facilitate comparisons of different HIFU settings and their effects on the immune system [68]. Recently, the first animal models for mechanical HIFU have been developed [8]. Some murine studies suggest that mechanical HIFU induces a stronger anti-tumor immune response than thermal HIFU [52, 53]. However, studies describing mechanical HIFU are limited, underscoring the need for further investigation. Whether sequential HIFU conditions exist that are sufficient to trigger potent immune responses in the absence of an adjuvant remains to be answered. Furthermore, it will be rewarding to look for the best HIFU ablation conditions that can optimally boost immunotherapy and synergize with immune adjuvants. Additionally, it will be important to determine the relative immunogenicity and nature of HIFU-created tumor debris, compared to for instance tumor debris after cryoablation or RFA.

In clinical practice, local recurrence of the primary tumor and/or emergence of pre-existing metastases are the main limitations of successful curative treatment using tumor ablation methods. Recent clinical results with current ablation treatments have shown that these problems persist with HIFU [10, 15]. To overcome these limitations, it will be key to consider combination therapies, combining ablation with adjuvants or checkpoint blockade therapy to generate strong systemic antitumor immunity for individual patients.

## Abbreviations

APC: Antigen-presenting cell
CEA: Carcinoembryonic antigen
CTL: Cytotoxic T lymphocyte
DAMP: Damage-associated molecular pattern
DC: Dendritic cell
HCC: Hepatocellular carcinoma
HIFU: High-intensity focused ultrasound
HSP: Heat-shock protein
LA: Laser ablation
LN: Lymph node
MHC: Major histocompatibility complex
MR: Magnetic resonance
MWA: Microwave ablation
PRR: Pattern recognition receptor
RFA: Radiofrequency ablation
TDLN: Tumor-draining lymph node
TLR: Toll-like receptor
VEGF: Vascular endothelial growth factor
